# Lavender Essential Oil Modulates Hepatic Cholesterol Metabolism in HepG2 Cells

**DOI:** 10.3390/cimb45010026

**Published:** 2023-01-03

**Authors:** Noemi Martella, Mayra Colardo, William Sergio, Michele Petraroia, Michela Varone, Daniele Pensabene, Miriam Russo, Sabrina Di Bartolomeo, Giancarlo Ranalli, Gabriella Saviano, Marco Segatto

**Affiliations:** 1 Department of Biosciences and Territory, University of Molise, Contrada Fonte Lappone, 86090 Pesche, Italy; 2 Department of Science, University Roma Tre, Viale Marconi 446, 00146 Rome, Italy

**Keywords:** cholesterol, borneol, essential oil, lavender, linalool, lipids, terpinen-4-ol

## Abstract

Cholesterol is an essential lipid that guarantees several biological processes in eukaryotic cells. Its metabolism is regulated by a complex protein network that could be significantly influenced by numerous exogenous sources, such as essential oils (EOs). For instance, it has been speculated that monoterpenoid and sesquiterpenoid compounds contained in lavender essential oil (LEO) may exert important hypocholesterolemic activities. However, the molecular mechanisms by which LEO influences cholesterol homeostasis are not characterized. In this work, we evaluated the ability of LEO to regulate the protein network that controls cholesterol metabolism in the HepG2 cell line. The main findings indicate that LEO administration increases intracellular cholesterol content. Concurrently, LEO affects the expression of proteins involved in cholesterol uptake, biosynthesis, and trafficking. These effects are partially mediated by terpinene-4-ol, one of the most abundant compounds in LEO. These results demonstrate that LEO modulates cholesterol metabolism in hepatic cells.

## 1. Introduction

Cholesterol plays both structural and functional roles that are essential for human biology. This steroid is a pivotal component of cell membranes, where it regulates the permeability and fluidity of the phospholipid bilayer. It is particularly concentrated in specialized sphingolipid-rich domains, such as rafts and caveolae. Cholesterol also represents the precursor for steroid hormones, vitamin D, and bile acids, being involved in a plethora of physiological processes [1,2]. Loss of cholesterol homeostasis is often associated with several genetic diseases, such as Smith–Lemli–Opitz syndrome (SLOS) and Niemann–Pick type C disease (NPC) [3,4]. In addition, cholesterol metabolism defects are involved in many other pathological conditions, including cardiovascular diseases (CVD), cancer, and neurodegeneration [1,5,6]. Given the importance of cholesterol in several physiological and pathological contexts, the body relies on a complex homeostatic network to modulate the availability of this lipid. The protein machinery that controls cholesterol levels is particularly active in the liver, where a significant portion of cholesterol metabolism occurs [5]. 

De novo cholesterol biosynthesis is guaranteed by the activity of the mevalonate (MVA) pathway, which involves more than 30 enzymatic reactions. The conversion of 3β-hydroxy-3β-methylglutaryl CoA (HMG-CoA) into MVA, operated by 3β-hydroxy-3β- methylglutaryl CoA reductase (HMGCR), is considered the key and rate-limiting step of cholesterol biosynthesis. Other relevant enzymatic reactions belonging to the MVA pathway are catalyzed by squalene epoxidase (SQLE) and squalene synthase (SQLS) [7].

In addition to de novo biosynthesis, cells may satisfy their cholesterol needs through the receptor-mediated endocytosis of lipoproteins. High-density lipoprotein uptake is mediated by scavenger receptor B1 (SR-B1), whereas low-density lipoprotein (LDL) uptake is assured by the expression of the LDL receptor (LDLr) and LDLr-related protein 1 (LRP1). The presence of apolipoproteins, such as apoE and apoB, ensures the binding of LDL to LDLr and LRP1 [8]. Once internalized, LDL-cholesteryl esters are hydrolyzed by lysosomal acid lipases to generate free cholesterol, which leaves the lysosomal compartment and is distributed to other cellular structures through Niemann–Pick type C1 (NPC1) and type C2 (NPC2) proteins [1]. The long-term regulation of cholesterol metabolism is promoted by transcription factors that are called sterol regulatory element-binding proteins (SREBPs). In particular, SREBP-2 isoform selectively induces the expression genes involved in cholesterol regulation [9]. When intracellular cholesterol levels are low, SREBP-2 is proteolytically processed to yield a soluble active fragment which enters the nucleus and binds to specific DNA sequences, which are called sterol regulatory elements (SREs), leading to the transcription of genes that codify for proteins and enzymes implicated in cholesterol biosynthesis and uptake, such as HMGCR, SQLE, SQLS, and LDLr [10,11]. Conversely, excess cholesterol suppresses the transcription of cholesterologenic genes, and the efflux of this sterol is enhanced by the activity of ATP-binding cassette (ABC) subfamilies, especially ABCA1 and ABCG5/8 [12,13].

As mentioned above, alterations in cholesterol levels are frequently associated with different pathological conditions [1]. Thus, the identification of novel natural and synthetic compounds that are able to influence cholesterol metabolism is a topic of considerable importance in biomedical research. Essential oils (EOs) are rich in monoterpenoid and sesquiterpenoid compounds that show convincing hypocholesterolemic properties [14]. For instance, it has been speculated that lavender EO (LEO) may contain several compounds that demonstrate promising modulatory activity on cholesterol levels. Accordingly, the dietary extract of *Lavandula angustifolia* reduces serum cholesterol in rats [15]. Another report corroborates this finding, demonstrating that linalool, a significant component of LEO, markedly decreased total cholesterol and LDL-cholesterol plasma levels [16]. Nonetheless, the molecular mechanisms by which LEO modulates cholesterol levels remain elusive and poorly characterized.

Starting from these premises, the aim of this work is to evaluate whether LEO can affect the protein network that controls cholesterol homeostasis. To reach this objective, we used the HepG2 cell line as an experimental model. This liver cell line expresses a wide variety of liver-specific metabolic functions and is considered a valuable model for the study of cholesterol metabolism [17].

## 2. Results

### 2.1. LEO Yield and Composition

*Lavandula angustifolia* flowers were harvested in central Italy (Rosciano, PE). They were hydrodistillated to obtain an essential oil characterized by a typical smell, in a yield of 3.6%, calculated by the initial weight of the sample used.

The GC-MS analysis identified 61 compounds, corresponding to 98.1% of the total area of the chromatogram [18].

Table 1 reports the main chemical components (>1.0%) of the EO in percentages; the compounds are listed according to their elution order on a Rtx^®^-5 Restek capillary column; complete identification was performed by calculating the experimental retention indices (Exp. RI) and comparing them with those found in the literature (Ref. RI) [19]. A set of analytical standards was also used: Linalool, Borneol, Terpinen-4-ol, Camphor and Lavender Oil (Merck Life Science, Milan, Italy).

GC-MS analysis confirmed the presence of Linalool (36%) as the major component and characterizing essence of *L. angustifolia*. Among the other constituent parts identified, the LEO characterized in this study showed high content levels of borneol (19.3%), 1,8-Cineole (9.0%), and terpinen-4-ol (6.8%). Moreover, the oxygenated monoterpenes component represents the most abundant class (87.5%).

### 2.2. LEO Increases Intracellular Neutral Lipids and Cholesterol Content in HepG2 Cells

To evaluate the influence of cholesterol metabolism using a hepatic cell culture experimental model, we first investigated the putative effects of LEO on intracellular neutral lipids and cholesterol content. Thus, HepG2 cells were treated with LEO at a concentration of 0.005% (*v/v*) for 24 h. Subsequently, Oil Red O (ORO) staining was performed to estimate the amount of neutral lipids (triglycerides and cholesteryl esters), as well as the lipid droplet morphology [20].

**Figure 1:**
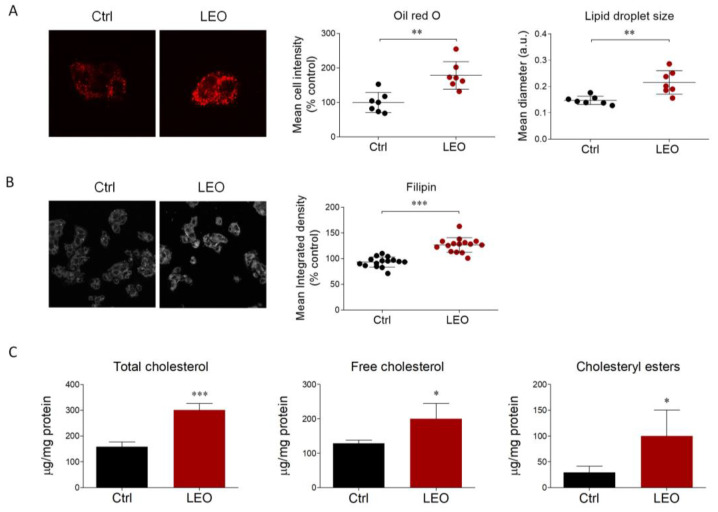
LEO increases the content of neutral lipids and cholesterol in HepG2 cells. (**A**) Representative images of Oil Red O staining (left panel), used to visualize intracellular neutral lipids on HepG2 cells treated with the vehicle (DMSO, Ctrl cells) or LEO (0.005%) for 24 h, and respective quantification of the mean cell intensity and the mean diameter of lipid droplets (right panel). *n* = 7 experimental replicates. (**B**) Representative images (left panel) and calculation of the mean integrated density (right panel) of filipin signal performed on HepG2 cells after treatments with the vehicle (Ctrl) and LEO (0.005%) for 24 h. *n* = 15 experimental replicates. (**C**) Quantification of intracellular cholesterol content (total cholesterol, free cholesterol, and cholesteryl esters) in vehicle- and LEO-treated HepG2 cells. *n* = 5 different experiments. Data represent means ± SD. Statistical analysis was assessed using the unpaired Student’s *t*-test. * *p* < 0.05; ** *p* < 0.01; *** *p* < 0.001.

Immunofluorescence analysis showed that LEO administration significantly increased the staining intensity and the size of lipid droplets, suggesting an accumulation of neutral lipids in HepG2 cells (Figure 1A). We then turned to filipin staining, demonstrating that LEO treatment promoted a buildup in intracellular free cholesterol (Figure 1B). This result was further corroborated by the quantitative colorimetric enzymatic assay, proving that total intracellular cholesterol was higher upon LEO stimulation. Interestingly, this effect was determined by a contributory increase in both free and esterified cholesterol (Figure 1C).

The data collected indicate that LEO significantly increases lipid accumulation, particularly in relation to cholesterol in its free and esterified forms.

### 2.3. LEO Modulates the Expression of Proteins and Enzymes That Control Cholesterol Metabolism

**Figure 2:**
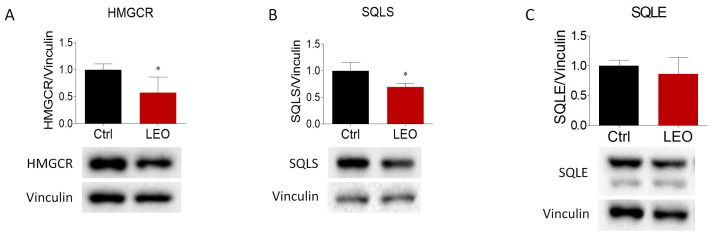
Effects of LEO administration on the main proteins involved in cholesterol biosynthesis. Representative Western blot and densitometric analysis of (**A**) HMGCR, (**B**) SLQS, and (**C**) SQLE proteins in HepG2 cells treated with the vehicle (DMSO, Ctrl cells) and LEO (0.005%) for 24 h. Vinculin was chosen as the loading control. *n* = 3 independent experiments. Data represent means ± SD. Statistical analysis was performed using the unpaired Student’s *t*-test. * *p* < 0.05.

Once we had assessed the impact of LEO administration on neutral lipids and cholesterol content, the proteins and enzymes that orchestrate cholesterol metabolism were analyzed to provide a mechanistic explanation. The key enzymes involved in cholesterol biosynthesis were assessed using a Western blot test. The protein expression of HMGCR and SQLS was significantly reduced after 24 h of LEO treatment (Figure 2A,B), whereas SQLE did not show any statistically significant change (Figure 2C). The reduction in HMGCR and SQLS suggests a decrease in cholesterol biosynthesis upon LEO stimulation.

**Figure 3:**
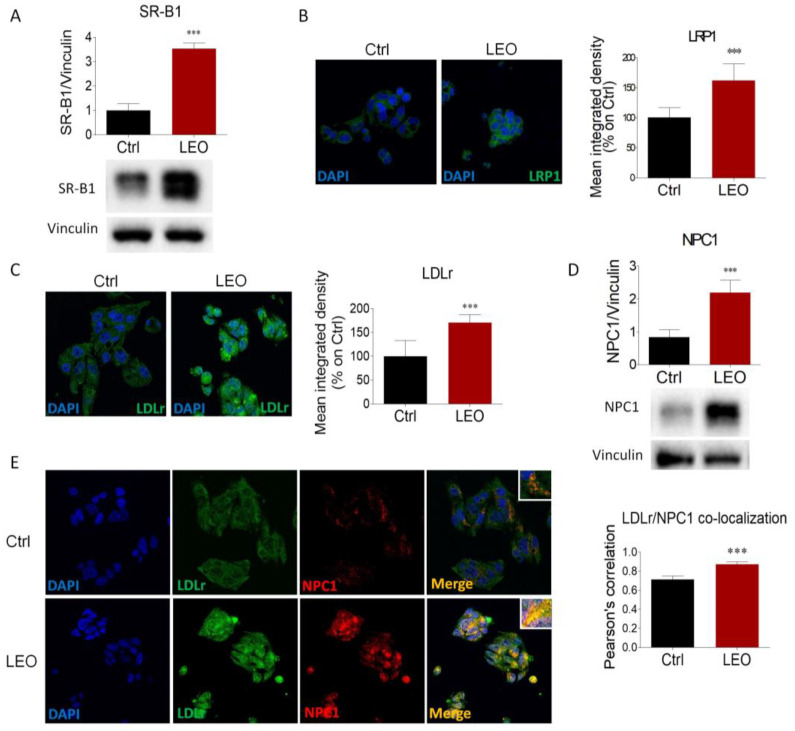
LEO treatment increases the expression of proteins involved in cholesterol uptake and intracellular trafficking in HepG2 cells. (**A**) Representative Western blot and densitometric analysis of SR-B1 in HepG2 cells treated with the vehicle (DMSO, Ctrl cells) and LEO (0.005%) for 24 h. Vinculin was chosen as the loading control. *n* = 3 independent experiments. Representative immunofluorescence and respective quantification of the mean fluorescence intensity of (**B**) LRP1 (green) and (**C**) LDLr (green) in HepG2 cells treated as in (A). DAPI (blue) was used to counterstain cell nuclei. *n* = 7 independent experiments. (**D**) Representative Western blot and densitometric analysis showing NPC1 expression in HepG2 cells treated as previously described. Vinculin served as the housekeeping protein. *n* = 4 biological replicates. (**E**) Co-immunofluorescence analysis was performed in HepG2 cells treated as previously reported, by using antibodies against NPC1 (red) and LDLr (green) (left panel). Nuclei were counterstained with DAPI. Pearson’s correlation coefficient *r* (right panel) was calculated to evaluate the protein co-localization. *n* = 7 different experiments. Data represent means ± SD. Statistical analysis was assessed using the unpaired Student’s *t*-test. *** *p* < 0.001.

Next, we evaluated the expression of proteins involved in lipoprotein uptake and intracellular cholesterol transport. Western blot analysis showed that expression of SR-B1, the main receptor involved in HDL uptake [21], was dramatically increased in LEO-treated HepG2 cells (Figure 3A), indicating a putative enhancement in HDL-cholesterol uptake. Morphological evaluation illustrated that LRP1 and LDLr immunoreactivities were remarkably higher after LEO treatment, suggesting that LDL uptake may be intensified (Figure 3B,C). The expression of NPC1, a resident endosomal/lysosomal protein involved in intracellular cholesterol trafficking [1], was also greatly increased (Figure 3D).

Upon lipoprotein binding, LDLr is internalized and trafficked along the endo-lysosomal compartment, where it can either be recycled to the surface of cell membrane for reuse or degraded by lysosomes [22]. Thus, co-localization of LDLr with endolysosomal proteins can be used as an index to assess the strength of cholesterol uptake. Confocal microscopy indicated that LDLr immunostaining was weak and diffuse throughout the cytoplasm in the control HepG2. On the contrary, LDLr immunopositivity in LEO-treated cells was more intense and strongly localized with NPC1, as demonstrated by the Pearson’s correlation index (Figure 3E). These results suggest that LEO administration significantly elicits cholesterol uptake and intracellular trafficking by increasing the expression of lipoprotein receptors and NPC1.

**Figure 4:**
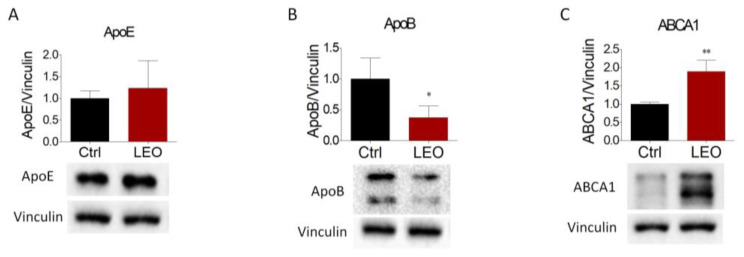
Effects of LEO on the expression of proteins involved in cholesterol transport in HepG2 cells. (**A**–**C**) After stimulation with LEO (0.005%) and the vehicle (DMSO, Ctrl cells) for 24 h, HepG2 cells were collected and subjected to Western blot analysis to evaluate apoE, apoB, and ABCA1 protein expression. Vinculin was chosen as the loading control. *n* = 3 independent experiments. Data represent means ± SD. Statistical analysis was performed using the unpaired Student’s *t*-test. * *p* < 0.05; ** *p* < 0.01.

Next, we evaluated the effects of LEO on the expression of proteins involved in cholesterol transport. We found that apoE and apoB are primarily synthetized in hepatic cells, where they are assembled into lipoproteins responsible for the shuttling of triglyceride and cholesterol from the liver to the peripheral tissues [23]. While apoE abundance did not change between the two experimental groups (Figure 4A), apoB expression was consistently reduced in LEO-treated cells (Figure 4B). In addition, ABCA1, a transporter involved in cholesterol efflux, was substantially increased following LEO stimulation (Figure 4C).

**Figure 5:**
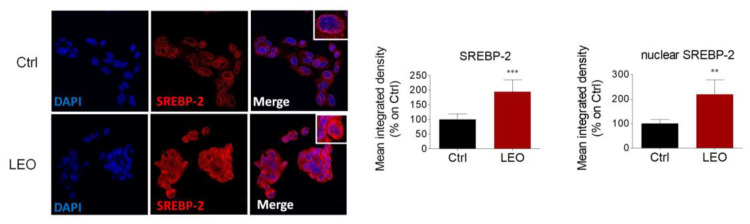
LEO induces SREBP-2 expression and nuclear translocation in HepG2 cells. Left panel: representative immunofluorescence of SREBP-2 (red) upon 24 h of LEO administration (0.005%). DAPI (blue) was used to counterstain the nuclei. Right panel: quantitative evaluation of the total and nuclear SREBP-2 protein fluorescence intensity. *n* = 7 different experiments. Data represent means ± SD. Statistical analysis was assessed using the unpaired Student’s *t*-test. ** p < 0.01; *** p < 0.001.

Given the profound alterations in cholesterol metabolism, we further evaluated whether LEO could affect SREBP-2, one of the most important transcription factors regulating cholesterol homeostasis [24]. Immunofluorescence analysis revealed that SREBP-2 immunoreactivity in control HepG2 cells is principally confined to the cytoplasm, with higher intensity mainly seen at the perinuclear level, suggesting subcellular localization to the endoplasmic reticulum and nuclear envelope. However, LEO treatment significantly increased the overall SREBP-2 immunoreactivity and enhanced its localization at a nuclear level. These data led us to speculate that LEO favors SREBP-2 expression and the production of its active nuclear fragment (Figure 5).

### 2.4. Terpinen-4-ol Partially Reproduces the Effects Induced by LEO on Cholesterol Metabolism

Monoterpenoid alcohols are among the main constituents of EOs. Interestingly, we revealed that terpinen-4-ol is one of the four most abundant compounds found in the LEO collected in our laboratories [18]. Terpinen-4-ol is also the main component of tea tree oil (*Melaleuca alternifolia*) [25]. Since tea tree oil strongly affects lipid content in hepatocytes [26], we speculated that terpinen-4-ol could be responsible for the modulatory effects on cholesterol metabolism exerted by LEO used in this study.

Thus, HepG2 cells were treated with terpinen-4-ol for 24 h, which was administered in the same dose (0.00034%) as present in LEO. Immunofluorescence analysis revealed that terpinene-4-ol significantly augmented ORO staining.

**Figure 6:**
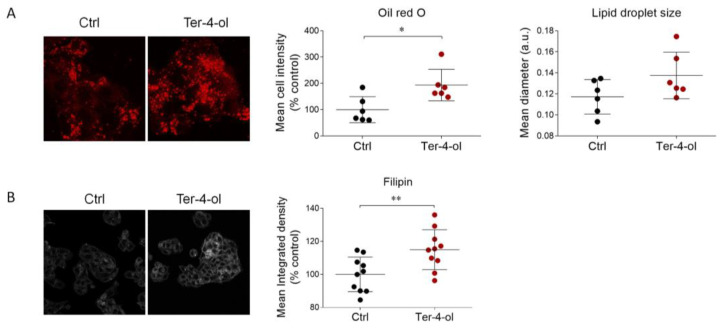
Effects of terpinen-4-ol on neutral lipids and free cholesterol. (**A**) Representative images (left) and respective quantification of the signal intensity and estimation of lipid droplet size (right) following Oil Red O staining performed on the vehicle- (DMSO) and terpinen-4-ol-treated (0.00034%) HepG2 cells for 24 h. *n* = 6 independent experiments. (**B**) Representative images and quantitative analysis of filipin staining detected in HepG2 cells stimulated as in (**A**). *n* = 10 different experiments. Data represent means ± SD. Statistical analysis was performed using the unpaired Student’s *t*-test. * p < 0.05; ** p < 0.01.

Concurrently, lipid droplet size was also increased, even though this parameter did not reach statistical significance (Figure 6A). Additionally, a buildup of free cholesterol, evaluated by filipin staining, was observed upon terpinen-4-ol treatment (Figure 6B).

**Figure 7:**
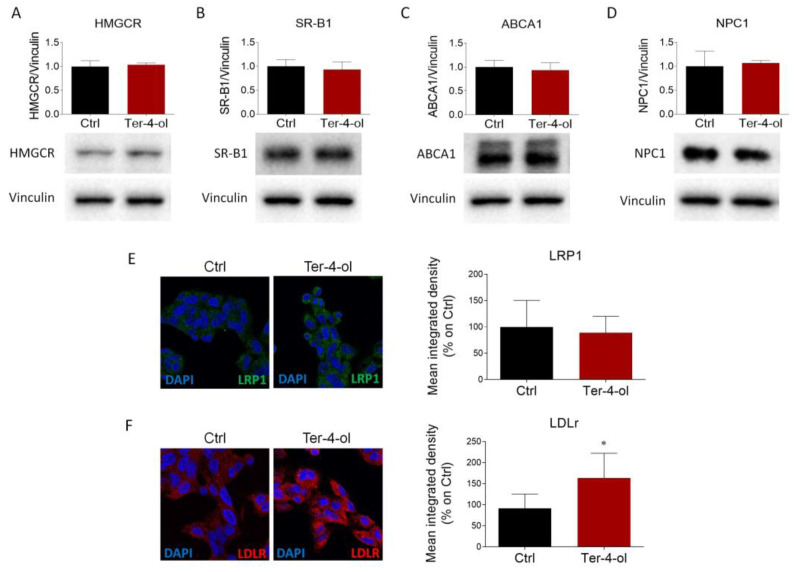
Effects of terpinen-4-ol on the main proteins that regulate cholesterol metabolism. (**A**–**D**). HepG2 cells were cultured in presence of vehicle (DMSO, Ctrl cells) or terpinen-4-ol (0.00034%) for 24 h and then evaluated by Western blot to assess HMGCR, SR-B1, ABCA1, and NPC1 protein expression. Vinculin was used as the loading control. *n* = 3 independent experiments. (**E**,**F**) Immunofluorescence and confocal analysis (left panel) for LDLr (red) and LRP1 (green) proteins performed in HepG2 cells after vehicle (Ctrl) and terpinen-4-ol (0.00034%) administration for 24 h. DAPI (blue) was used to counterstain the nuclei. Graphs (right panel) represent the respective signal intensity quantification of both receptors. *n* = 6 different experiments. Data represent mean ± SD. Statistical analysis was assessed using the unpaired Student’s *t*-test. * p < 0.05.

This compound did not affect the expression levels of the main proteins involved in cholesterol metabolism, such as HMGCR, SR-B1, ABCA1, NPC1, and LRP1 (Figure 7A–E). Conversely, terpinen-4-ol treatment similarly increased LDLr abundance to the levels previously observed following LEO administration (Figure 7F).

Our results indicate that terpinen-4-ol partially mimics the effects induced by LEO when evaluated as a whole. Specifically, this monoterpenoid promotes cholesterol accumulation, which is likely due to the increased LDLr expression.

### 2.5. Linalool and Borneol Do Not Influence Cholesterol Metabolism in HepG2 Cells

The data collected in this study indicate that terpinen-4-ol only partially mediates the effects observed upon LEO treatment. Thus, we focused our attention on linalool and borneol, since they represent the most abundant compounds in the LEO employed in this study [18]. Furthermore, both linalool and borneol were already identified as pivotal modulators of lipid metabolism [16,27,28].

**Figure 8:**
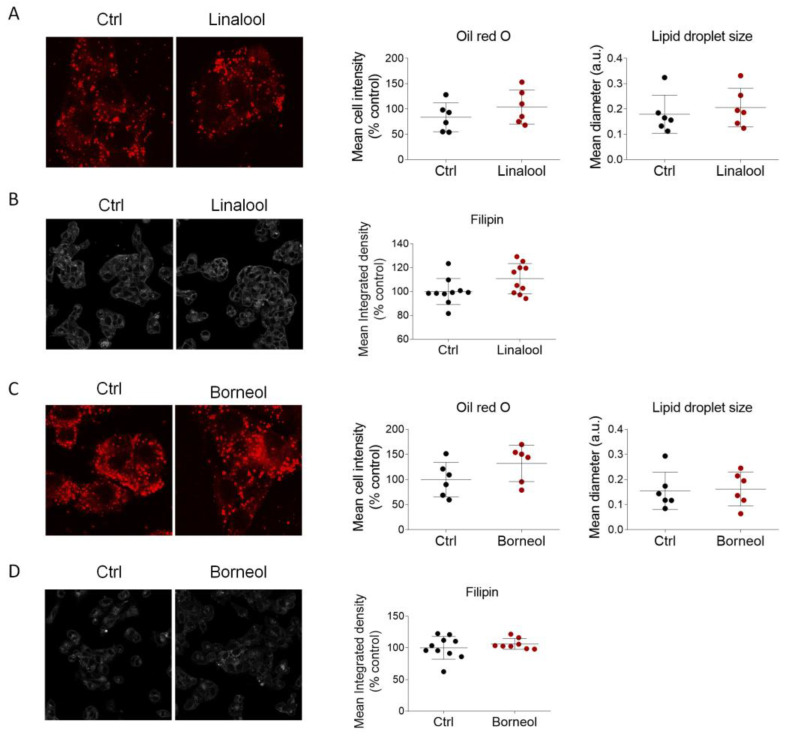
Linalool and borneol do not modulate the content of neutral lipids and cholesterol in HepG2 cells. (**A**) Representative images (left panel) and respective densitometric analysis (right panels) of Oil Red O staining in HepG2 cells treated with the vehicle (DMSO, Ctrl cells) and linalool (0.0018%) for 24 h. *n* = 6 different biological replicates. (**B**) Filipin staining and its respective fluorescence intensity in HepG2 cells treated as in (**A**). *n* = 10 different experiments. (**C**) Representative images (left panel) and respective densitometric analysis (right panels) of Oil Red O staining in HepG2 cells treated with the vehicle (Ctrl) and borneol (0.00097%) for 24 h. *n* = 6 different biological replicates. (**D**) Filipin staining and its respective fluorescence intensity in HepG2 cells treated as in (**C**). *n* = 10 different experiments. Data represent mean ± SD and the unpaired Student’s *t* test was used for the statistical analysis.

Our results highlighted that linalool, at the same dose as present in LEO (0.0018%), modified neither the intensity of ORO staining and lipid droplet size (Figure 8A), nor the free cholesterol content as evaluated by filipin staining (Figure 8B). As such, no changes were observed in the protein network that controls cholesterol metabolism, such as HMGCR, SR-B1, ABCA1, NPC1, LDLr and LRP1 (Supplementary Figure S1A–F).

Similarly, borneol, at the same dose as present in LEO (0.00097%), was ineffective in modulating the accumulation of neutral lipids and free cholesterol in HepG2 cells (Figure 8C,D), as well as the proteins involved in cholesterol regulation (Supplementary Figure S2A–F).

## 3. Discussion

Cholesterol is a component that is essential to all eukaryotic cells and exerts crucial biological functions, ranging from structural roles to signal transduction activities. Cholesterol homeostasis is guaranteed by a delicate balance between biosynthesis and uptake, and alterations in the network controlling its metabolism can easily lead to pathological alterations [1]. For these reasons, the discovery of new methods for modulating cholesterol metabolism are attracting increasing interest in biomedical research. Over the last few decades, several naturally occurring compounds have been investigated for their potential influence on cholesterol metabolism, particularly in the context of CVD and liver dysmetabolism [14]. For instance, some reports suggest that LEO may exert hypolipidemic effects, but the molecular mechanisms of this activity are mostly unknown.

Thus, the aim of this work was to assess the ability of LEO to modulate cholesterol metabolism in a hepatic cell line.

Our results demonstrate that LEO elicits profound alterations in lipid homeostasis, as it promotes the accumulation of neutral lipids and total, free, and esterified cholesterol. These effects are accompanied by alterations in the expression of the main proteins belonging to cholesterol regulatory network. The LEO-induced buildup of cholesterol is likely due to the upregulation of lipoprotein uptake, as suggested by the dramatic increase in SR-B1, LDLr, and LRP1. The induction of these lipoprotein receptors could be mediated, at least in part, by the increased activation of SREBP-2, whose nuclear translocation is enhanced upon LEO administration. Notably, SREBP-2 regulates the transcription of several genes that control cholesterol metabolism. In this context, it is well-known that LDLr and SR-B1 can be directly regulated by SREBP-2 because of the presence of sterol regulatory element (SRE) DNA sequences on their genes [29,30]. Additionally, other findings highlight that SREBP-2 is responsible for the upregulation of LRP1 expression in HepG2 and Hep3B liver cell lines [31]. The induction of lipoprotein receptors by LEO supports the hypocholesterolemic properties of *Lavandula angustifolia* already reported in literature. Specifically, oral administration of *Lavandula angustifolia* extract for 25 days led to a reduction in serum cholesterol, LDL, and VLDL [15]. In this context, our data allow us to speculate that a reduction in plasma cholesterol can be achieved by increased cellular uptake of lipoproteins from the circulation.

Concurrently, LEO decreases the expression of HMGCR and SQLS, two main enzymes involved in cholesterol biosynthesis. This effect could be mediated by the induction of a negative feedback mechanism induced by the buildup of intracellular cholesterol derived from lipoprotein uptake. In keeping with this notion, it is well established that high intracellular sterol levels induce HMGCR binding to insulin-induced gene (Insig) proteins, which mediate the ubiquitylation of the enzyme and its subsequent proteasomal-dependent degradation [32,33]. Interestingly, a similar mechanism has recently been described for SQLS [34]. In this work, we reported a significant rise in ABCA1 expression. This transporter is mainly involved in cholesterol efflux; thus, it can be hypothesized that LEO treatment leads to an increase in ABCA1 levels with the attempt to counteract excessive intracellular cholesterol accumulation. LEO-mediated ABCA1 expression is in line with the enhanced activation of SREBP-2. Indeed, it has been shown that SREBP-2 positively triggers ABCA1 transcription by enabling the production of oxysterol ligands for LXR [35]. 

In attempting to identify the specific compounds responsible for the effects induced by LEO, we tested the ability of linalool, borneol, and terpinen-4-ol to modulate cholesterol metabolism. These three monoterpene alcohols are the most abundant compounds found in the LEO used in this study; intriguingly, they have already been identified as putative cholesterol modulators. For instance, it has been reported that linalool significantly reduces plasma cholesterol in preclinical rodent models. Mechanistically, plasma cholesterol reduction is associated with increased LDLr expression and reduced HMGCR levels because of the enhanced degradation of the enzyme [16,36]. Borneol significantly reduces circulating total cholesterol, LDL, and VLDL in streptozotocin-induced diabetic rats [28]. Other cholesterol-lowering properties were described for tea tree oil, which is particularly rich in terpinen-4-ol [26]. Our results indicate that linalool and borneol do not exert any effect on the parameters taken into consideration in this study, thus excluding their involvement in the alterations induced by LEO. On the other hand, terpinen-4-ol only partially reproduces the biological activity mediated by LEO on cholesterol metabolism; in particular, terpinen-4-ol elicits the accumulation of neutral lipids and free cholesterol, and this effect is sustained by increased LDLr expression. On the other hand, terpinen-4-ol administration does not mimic the influence of LEO on other proteins and enzymes belonging to cholesterol regulatory network, suggesting that other minor compounds occurring in LEO may concur with the regulatory activities observed in this study.

In conclusion, this work elucidates, for the first time, the molecular mechanisms linking LEO and the regulation of cholesterol homeostasis. Even though these data provide some new clues about the biological activity of LEO, more efforts should be made to clearly identify the putative molecules responsible for cholesterol modulation. In addition, such effotrs would allow us to better comprehend the biological consequences of LEO exposure. Indeed, while compounds capable of increasing lipoprotein receptor expression result in a cholesterol-lowering effect, excessive uptake could lead to harmful lipid intracellular accumulation. Our work might suggest the establishment of this circumstance, since LEO-treated HepG2 resembles a cellular phenotype associated with hepatic steatosis.

## 4. Materials and Methods

### 4.1. Plant Material

*L. angustifolia* flowers were harvested in September 2020 during the balsamic period, at Villa Vanda Farm, Rosciano, Abruzzo Region, Italy (42°20′59.83″ N, 14°01′54.88″ E). The plant was identified by Dr. Fortini, and a voucher specimen was deposited in the Herbarium of DiBT, University of Molise.

### 4.2. Isolation of the Essential Oil

Fresh flowers (200 g) of *L. angustifolia* were hand selected, cleaned, and then subjected to hydrodistillation for 3 h according to the standard procedure described in the Council of Europe [37]. The EO was dried over anhydrous sodium sulfate to remove traces of water and then stored in dark vials at 4 °C prior to GC-MS analysis.

### 4.3. GC-FID/GC-MS Analysis

The analysis of LAO was performed on a Trace GC Ultra (Thermo Fisher Scientific) gas chromatography instrument equipped with a Rtx^®^-5 Restek capillary column (30 m × 0.25 mm i.d., 0.25 μm film thickness) and coupled with an ion-trap (IT) mass spectrometry (MS) detector Polaris Q (Thermo Fisher Scientific, Waltham, MA, USA).

A programmed temperature vaporizer (PTV) injector and a PC with a chromatography station Xcalibur (Thermo Fisher Scientific, Waltham, MA, USA) were used. The ionization voltage was 70 eV; the source temperature was 250 °C; full scan acquisition in positive chemical ionization was from m/z 40 up to 400 a.m.u. at 0.43 scan s^−1^. The column temperature was maintained at 40 °C for 5 min, then programmed to increase to 250 °C at a rate of 3 °C/min and held, by using an isothermal process, for 10 min; the carrier gas was He (1.0 mL/min); 1 μL of each sample was dissolved in *n*-hexane (1:500 *n*-hexane solution) and injected. The experiment was repeated three times.

### 4.4. Identification of Essential Oil Components

Components were identified by comparison of their mass spectra fragmentation patterns with the NIST 02, Adams, and Wiley 275 mass spectral libraries [38,39] and by comparing their retention index calculated against a homologous series of *n*-alkanes (C8–C20) [40,41].

The average value of relative contents (%) of the sample components were computed from peak areas obtained in triplicate without any corrections [42].

### 4.5. Cells Cultures

HepG2 cells were cultured at 5% CO_2_ in a DMEM medium at high glucose, containing 10% (*v/v*) fetal bovine serum (FBS), L-glutamine (2 mM), with the addition of penicillin/streptomycin solution. For all experiments, 200.000 cells per well were seeded. Five hours after seeding, the HepG2 cells were treated with lavender essential oil (0.005%), extracted as previously described, and treated with the single molecules under study. Based on the chemical-analytical characterization of the oil and the abundance of each compound in the essential oil we extracted, the individual terpenoids were tested as follows: linalool (74856, Merck Life Science, Milan, Italy) 0.0018%; borneol (420247, Merck Life Science, Milan, Italy) 0.00097%; terpinen-4-ol (86477, Merck Life Science, Milan, Italy) 0.00034%. In order to facilitate solubilization in the growth medium, the appropriate amount of oil and the individual compounds were first conveyed into FBS (at a final concentration of 10% in DMEM) and then into DMEM. Control cells received DMSO (dilution 1:1000 in cell culture medium) as the vehicle.

### 4.6. Oil Red O Staining and Quantification

Oil Red O staining was performed as previously reported [24]. Briefly, HepG2 cells were seeded on coverslips and treated with the different compounds under study, as previously described. After 24 h, cells were fixed in paraformaldehyde (4% solution) for 10 min and rinsed gently three times with PBS. The fixed cells were then incubated with 60% isopropanol for 5 min and then stained with 1 mL of Oil Red O working solution (O1391-250ML, Merck Life Science, Milan, Italy) for 15 min at room temperature while shaking. After incubation with the staining solution, cells were washed five times with distilled water to remove excess staining. Finally, coverslips were mounted with the Fluoroshield mounting medium (F6182, Merck Life Science, Milan, Italy) and the autofluorescent probe was visualized by confocal microscopy (TCS SP8; Leica Microsystems, Buccinasco, Italy). Images were acquired using the Leica TCS SP8 microscope equipped with 63× magnification and Leica LAS X software (Leica Microsystems, Buccinasco, Italy). Both the Oil red O intensity quantification and the measurement of the mean diameter of lipid droplets were performed using ImageJ software (National Institutes of Health, Bethesda, MD, USA) for Windows.

### 4.7. Filipin Staining and Quantification

Filipin staining was performed as previously described [43], using Filipin complex (F9765, Merck Life Science, Milan, Italy). Filipin stock solution (10 mg/mL in PBS) was prepared at the time of use. Cells were fixed in paraformaldehyde (4% solution) for 10 min and then washed with PBS. Then, the cells were immediately stained with 1 mL of filipin working solution (0.05 mg/mL in PBS) for 2 h at room temperature in the dark. Next, the cells were washed three times with PBS and the coverslips were mounted with the Fluoroshield mounting medium (F6182, Merck Life Science, Milan, Italy) and immediately observed under a confocal microscope (TCS SP8; Leica Microsystems, Wetzlar, GermanyBuccinasco, Italy) equipped with UV filters (excitation 340–380 nm). Images were acquired at 40× magnification. Filipin quantification was calculated as the mean fluorescence intensity per cell area using ImageJ software (National Institutes of Health, Bethesda, MD, USA) for Windows.

### 4.8. Cholesterol Quantification

Cholesterol quantification was performed using an enzymatic colorimetric assay (Cholesterol Quantitation Kit, MAK043, Merck Life Science, Milan, Italy) following the manufacturer’s instructions.

### 4.9. Lysate Preparation and Western Blot Analysis

HepG2 cells were lysed in 30 μL of lysis buffer (0.125 M Tris-HCl containing 10% SDS, protease inhibitor cocktail, pH 6.8) by sonication (duty cycle 20%, output 3) for 30 s in order to obtain a total lysate. The samples were then centrifuged at 10,000 *g* for 10 min to remove cellular debris. Lowry’s method was used to assess the protein concentration. Then, Laemmli buffer was added, and the samples were boiled at 95 °C for 3 min. Protein extracts (twenty micrograms of protein) were resolved on SDS-PAGE, and the transfer onto nitrocellulose membrane was performed using a turbo trans-blot transfer system (Biorad Laboratories, Milan, Italy). Subsequently, the membrane was incubated at room temperature for 1 h with 5% fat-free milk powder in Tris-buffered saline (25 mM Tris-HCl, 138 mM NaCl, 27 mM KCl, 0.05% Tween-20, pH 6.8) and probed overnight at 4 °C with the following primary antibodies: anti-ABCA1 (Santa Cruz Biotechnology, Dallas, TX, USA, sc-58219, dilution 1:200), anti-ApoB (Santa Cruz Biotechnology, Dallas, TX, USA, sc-13538, dilution 1:500), anti-ApoE (Santa Cruz Biotechnology, Dallas, TX, USA, sc-390925, dilution 1:400), anti-GAPDH (Santa Cruz Biotechnology, Dallas, TX, USA, sc-32233, dilution 1:5000), anti-HMGCR (Abcam, Cambridge, UK, ab242315, 1: 1000 dilution), anti-NPC1 (Novus Biologicals, NB400-148, dilution 1:3000), anti-SQLE (Santa Cruz Biotechnology, Dallas, TX, USA, sc-271651, dilution 1:1000), anti-SQLS (Santa Cruz Biotechnology, Dallas, TX, USA, sc-271602, dilution 1:500), anti-SR-B1 (Abcam, Cambridge, UK, ab52629, dilution 1:500), and anti-Vinculin (Santa Cruz Biotechnology, Dallas, TX, USA, sc-73614, dilution 1:5000). Next, the membranes were probed for 1 h with horseradish peroxidase-conjugated secondary IgG antibodies (Bio-Rad Laboratories, Milan, Italy). Protein-antibody immunocomplexes were visualized using clarity ECL Western blotting (Bio-Rad Laboratories, Milan, Italy, No. 1705061), and chemiluminescence was recorded using the ChemiDoc MP system (Bio-Rad Laboratories, Milan, Italy). The obtained images were subjected to densitometric analysis using ImageJ software (National Institutes of Health, Bethesda, MD, USA) for Windows. All samples were normalized for protein loading with Vinculin or GAPDH, which were chosen as housekeeping proteins. Densitometric calculations were derived from the ratio between the protein band intensity and the respective housekeeping protein.

### 4.10. Immunofluorescence Staining

Immunofluorescence was performed following the protocol previously described [44]. Cells were fixed in paraformaldehyde (4% in PBS) and probed overnight at 4 °C with the following antibodies: anti-LDLr (Santa Cruz Biotechnology, sc-11824, dilution 1:50), anti-LRP1 (Santa Cruz Biotechnology, Dallas, TX, USA, sc-25469, dilution 1:50), anti-NPC1 (Novus Biologicals, NB400-148, dilution 1:100), and anti-SREBP2 (Santa Cruz Biotechnology, Dallas, TX, USA, sc-13552, dilution 1:50). Subsequently, HepG2 cells were incubated for 1 h at room temperature with donkey anti-goat secondary antibody Alexa Fluor 488 (ThermoFisher Scientific, Milan, Italy, A-11055), rabbit anti-goat secondary antibody Alexa Fluor 555 (ThermoFisher Scientific, Milan, Italy, A-27017), goat anti-mouse secondary antibody Alexa Fluor 555 (ThermoFisher Scientific, Waltham, MA, USA, A28180), and goat anti-rabbit secondary antibody Alexa Fluor 488 (ThermoFisher Scientific, Waltham, MA, USA, A27034). The cell nuclei were counterstained with DAPI and, finally, coverslips were mounted with Fluoroshield mounting medium (F6182, Merck Life Science, Milan, Italy). The preparations were examined under confocal microscopy (TCS SP8; Leica, Wetzlar, Germany), as described above.

### 4.11. Statistical Analysis

All the results presented in this study were expressed as means ± SD (standard deviation) and the unpaired Student’s *t*-test was used to compare the experimental groups. *p* < 0.05 was considered to indicate a statistically significant difference. Statistical analysis was carried out using GraphPad Prism 5 (GraphPad, La Jolla, CA, USA) for Windows.

## Figures and Tables

**Table 1:** Main chemical composition (>1%) of the essential oils (EOs) extracted from the flowers of *L. angustifolia*. L. AMO—aliphatic monoterpenoids; BMO—bi-and tricyclic monoterpenoids; AS—aliphatic sesquiterpenes; ASO—aliphatic sesquiterpenoids; MSO—monocyclic sesquiterpenoids; OT—others. SD—standard deviation; Exp. RI—experimental retention index; Ref. RI—literature data.

Compound	Exp. RI	Ref. RI	LEO Area % ± SD	Abbr.
1,8-Cineole	1034	1031	9.00 ± 0.29	BMO
Linalool oxide cis	1075	1072	1.68 ± 0.04	AMO
Linalool oxide trans	1089	1086	1.49 ± 0.02	AMO
Linalool	1105	1096	36.03 ± 0.10	AMO
Camphor	1148	1146	6.80 ± 0.07	BMO
Borneol	1170	1169	19.35 ± 0.12	BMO
Terpinen-4-ol	1182	1177	6.81 ± 0.02	BMO
Exil butirate	1196	1192	1.17 ± 0.02	OT
Linalyl acetate	1262	1257	2.75 ± 0.02	AMO
β-Farnesene	1462	1456	1.50 ± 0.01	AS

## Data Availability

Not applicable.

## Supplementary Materials

The following supporting information can be downloaded at: https://www.mdpi.com/article/10.3390/cimb45010026/s1, Figure S1: Linalool administration does not alter the expression of the main proteins and enzymes of cholesterol homeostasis. Figure S2: Borneol (0.00097%) treatment for 24 h does not influence the expression of proteins belonging to the cholesterol regulatory network.
