# Supplementary material for: Lavender Essential Oil Modulates Hepatic Cholesterol Metabolism in HepG2 Cells
Source: Curr Issues Mol Biol. 2023 Jan 3;45(1):364–78. doi: 10.3390/cimb45010026 (PMC9857966; doi:10.3390/cimb45010026)
Supplement: Supplementary file 1 [file cimb-45-00026-s001.zip › cimb-2127975-supplementary.pdf]

## Supplementary Materials

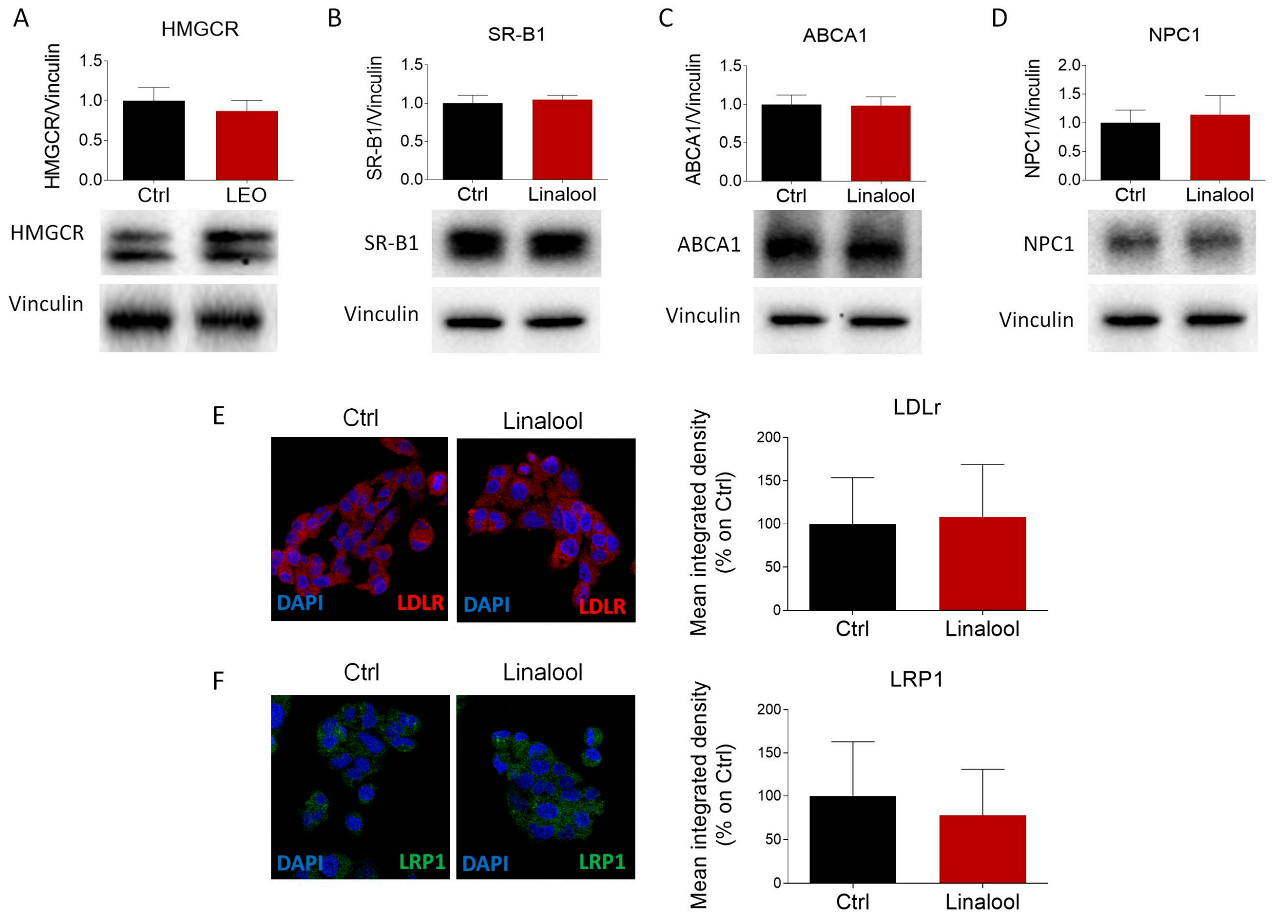


**Figure S1**. *Linalool administration does not alter the expression of the main proteins and enzymes of cholesterol homeostasis.* (***A-D***) HepG2 cells were cultured in presence of vehicle (Ctrl) or linalool (0.0018%) for 24 hours. Subsequently, Western blot and densitometric analysis of HMGCR, SR-B1, ABCA1 and NPC1 proteins was carried out. n = 3 independent experiments. Vinculin served as a housekeeping protein to normalize protein loading. (**E-F**) Immunofluorescence and confocal analysis for LDLr (red) and LRP1 (green) protein (left panel) and mean integrated density evaluation (right panel) in HepG2 cells. Nuclei were counterstained with DAPI. *n* = 6 different experiments. Data represent means ± SD. Statistical analysis was performed by using unpaired Student's t-test.

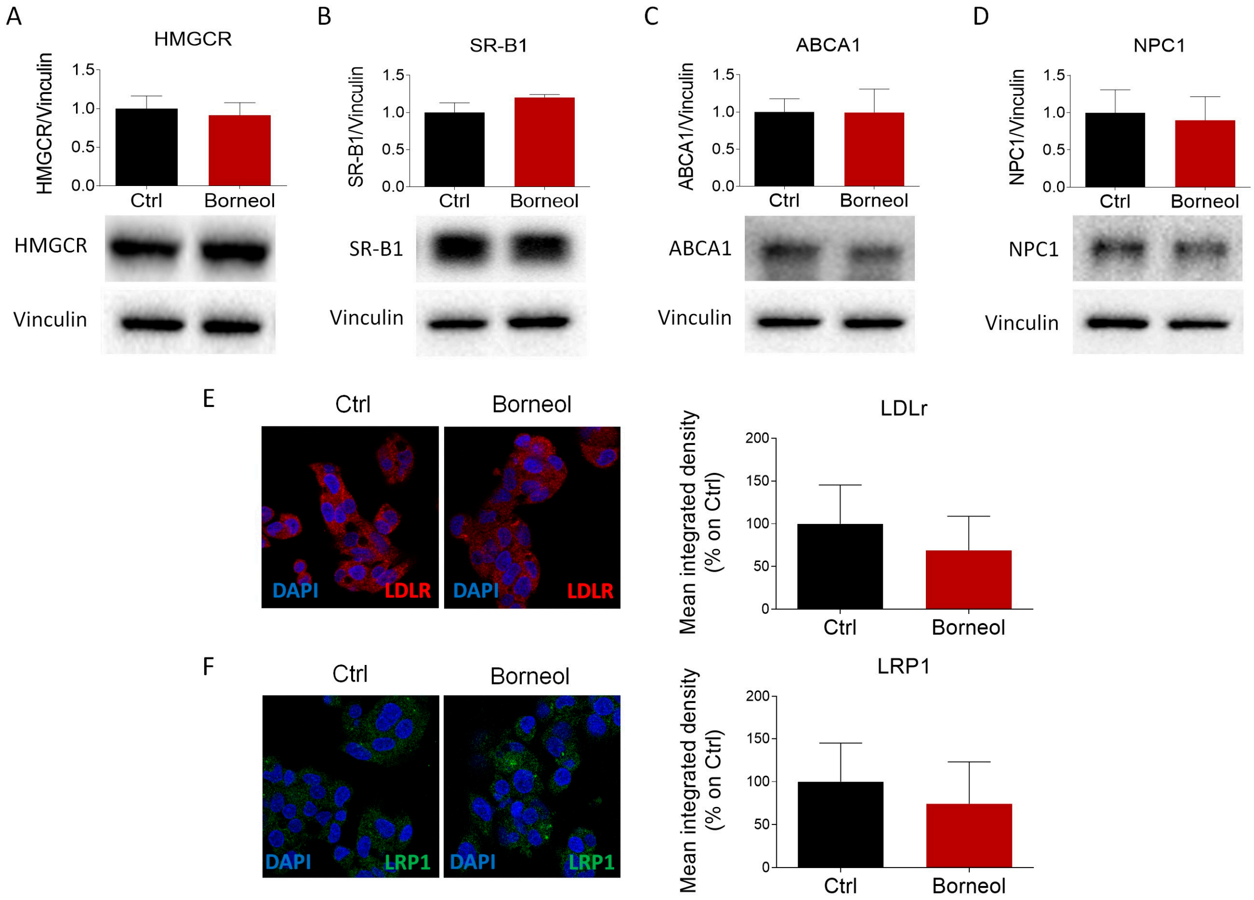


**Figure S2**. *Borneol (0.00097%) treatment for 24 hours does not influence the expression of proteins belonging to cholesterol regulatory network.* (**A-D**) Representative Western blot and densitometric analysis of HMGCR, SR-B1, ABCA1 and NPC1 proteins. Vinculin was chosen as loading control. n = 3 independent experiments. (**E-F**). Representative immunofluorescence (left panel) and relative quantification of LDLr (red) and LRP1 (green) intensity (right panel). Nuclei were counterstained with DAPI. n = 6 different experiments. Data represent means ± SD. Statistical analysis was performed by using unpaired Student's t-test.
